# A hybrid model for predicting response to risperidone after first-episode psychosis

**DOI:** 10.47626/1516-4446-2024-3608

**Published:** 2025-01-22

**Authors:** Giovany Oliveira Costa, Vanessa K. Ota, Matheus Rodrigues Luiz, Joice Santos Rosa, Gabriela Xavier, Jessica Honorato-Mauer, Marcos L. Santoro, Carolina Muniz Carvalho, Daniel A. Cavalcante, Amanda V.G. Bugiga, Rodrigo A. Bressan, Gerome Breen, Ary Gadelha, Cristiano Noto, Diego R. Mazzotti, Sintia I. Belangero

**Affiliations:** 1 Laboratório de Neurociências Integrativas (LiNC), Escola Paulista de Medicina, Universidade Federal de São Paulo (UNIFESP-EPM), São Paulo, SP, Brazil; 2 Disciplina de Genética, Departamento de Morfologia e Genética, UNIFESP-EPM, São Paulo, SP, Brazil; 3 Departamento de Psiquiatria, UNIFESP-EPM, São Paulo, SP, Brazil; 4 Departamento de Bioquímica, UNIFESP-EPM, São Paulo, SP, Brazil; 5 Social, Genetic and Developmental Psychiatry Centre, King’s College London, London, UK; 6 Institute of Psychiatry, Psychology and Neuroscience, London, UK; 7 Department of Internal Medicine, University of Kansas Medical Center, Kansas City, KS, USA

**Keywords:** Mental disorders, antipsychotic agents, risperidone, machine learning, genes

## Abstract

**Objective:**

Patient response to antipsychotic drugs varies and may be related to clinical and genetic heterogeneity. This study aimed to determine the performance of clinical, genetic, and hybrid models to predict the response of patients in first-episode psychosis (FEP) to the antipsychotic risperidone.

**Methods:**

We evaluated 141 antipsychotic-naive FEP patients before and after 10 weeks of risperidone treatment. Patients who had a response rate equal to or higher than 50% on the Positive and Negative Syndrome Scale (PANSS) were considered responders (n=72; 51%). Analyses were performed using a support vector machine (SVM), k-nearest neighbors (kNN), and random forests (RF). Clinical and genetic (with single-nucleotide variants [SNVs]) models were created separately. Hybrid models (clinical + genetic factors) with and without feature selection were created.

**Results:**

Clinical models presented greater balanced accuracy (63.3%; CI 0.46-0.69) with the SVM algorithm than the genetic models (balanced accuracy: 58.5% [CI 0.41-0.76], kNN algorithm). The hybrid model, which included duration of untreated psychosis, Clinical Global Impression-Severity (CGI-S) scale scores, age, cannabis use, and 406 SNVs, showed the best performance (balanced accuracy: 72.9% [CI 0.62-0.84], RF algorithm).

**Conclusion:**

A hybrid model including clinical and genetic predictors can enhance prediction of response to antipsychotic treatment.

## Introduction

The first episode of psychosis (FEP) usually occurs between late adolescence and early adulthood,^1^ and early onset is associated with a severe disease course.^2^ This critical period presents unique opportunities for secondary prevention, and a delay in treatment after FEP can lead to very poor prognosis.^3^

Approximately 80% of individuals experience recurrence in the first 5 years after FEP, mainly due to treatment discontinuation.^4^ The response to pharmacotherapy has a major impact on reducing symptoms in the first 2 weeks.^5^ However, approximately 20-60% of patients do not respond adequately,^6^,^7^ and only one in seven patients experiences complete symptom improvement.^8^

Previous studies have investigated reliable clinical variables capable of predicting the response to treatment with antipsychotics, namely nonmodifiable factors^9^ such as biological sex,^10^ age of onset, and symptom severity,^2^ as well as modifiable factors such as the duration of untreated psychosis (DUP).^11^ Single-nucleotide variants (SNVs) have also been associated with individual response to drug treatment.^12^ SNVs, including expression quantitative trait loci (eQTL), are associated with changes in the expression of pharmacogenes (i.e., those involved in drug metabolism).^13^

Studies have focused on genes associated with antipsychotic pharmacokinetics, such as cytochrome P450 family 2 subfamily D member 6 (*CYP2D6*),^14^ and pharmacodynamics, such as genes related to neurotransmitter pathways.^15^ It is still unclear whether the mechanism of treatment response observed in studies is associated with pharmacogenes, treatment status, or their interactions in chronically treated patients.^16^ Therefore, the inclusion of antipsychotic-naive FEP patients in various analyses is extremely advantageous, since this would allow assessment of early response in the absence of chronic exposure to antipsychotic drugs, which could alter receptor expression and brain morphology. However, longitudinal studies – particularly pharmacogenetic studies – are lacking. Precision psychiatry has implemented machine learning models that utilize multiomic, clinical, neuroimaging, and genetic datasets to develop tools capable of predicting responses to different interventions, with the potential for future clinical application. Thus, integration of clinical and genetic data of patients with FEP has the potential to generate robust models with increased performance to predict treatment response. Therefore, this study aimed to determine the performance of a combination of clinical and genetic factors in predicting the response of patients in FEP to the antipsychotic drug risperidone and identify which predictors contribute the most to treatment response.

## Methods

### First-episode psychosis cohort

The enrolled patients were part of a cohort of antipsychotic-naive FEP patients who were admitted to a public psychiatric emergency unit in the city of São Paulo, Brazil.

### Casuistic

We included 141 patients aged 16 to 35 years who had not previously taken antipsychotic drugs and whose psychotic episode was not attributable to an organic cause (intellectual disability, history of seizures, or brain tumor). Benzodiazepines were administered at the time of admission. Diagnoses were made by trained psychiatrists, based on the Structured Clinical Interview I (SCID-I) according to the DSM-IV criteria. All patients were evaluated at baseline and blood samples were collected. Risperidone was prescribed and patients were followed up for 73.7±29.0 days (approximately 10 weeks). Patients were divided into two groups – responders (n=72) and nonresponders (n=69) – according to their Positive and Negative Syndrome Scale (PANSS) scores.

### Clinical assessments

Trained psychiatrists performed clinical evaluations. Diagnoses were made according to SCID-I at each time point. The following data were collected at baseline and follow-up:

1. Sociodemographic data – biological sex, age, and household income.
2. Risk factors for mental disorders – family history of psychosis (report of at least one relative up to the third degree) and migration history (at least one change in city of residence during lifetime).
3. Psychiatric scales – Clinical Global Impression-Severity scale (CGI-S), Global Assessment of Functioning (GAF) scale, and PANSS.
4. Nicotine dependence – assessed using the Fagerström Test for Nicotine Dependence (FTND) and cannabis abuse/dependence, assessed with the Addiction Severity Index (ASI-6), a structured interview that evaluates past and current (last 30 days) use.
5. DUP – obtained from an adaptation of the Interview for the Retrospective Assessment of the Onset of Schizophrenia (IRAOS) instrument, determined as the time (in days) between the date of onset of psychotic symptoms and the date on which risperidone was started.^11^

The duration of treatment (in days) was the time elapsed between the date of initiation of antipsychotic treatment and the date of the second psychiatric evaluation. The response rate (%) was calculated using the PANSS score, according to the following formula.^17^ Individuals who presented a response rate of ≥ 50% were considered responders and those who presented a response rate of < 50% were nonresponders.

$$response\;rate\;(\%)=100\frac{\left(PANSS\;baseline-30)-(PANSS\;followup-30\right)}{\left(PANSS\;baseline-30\right)}$$

### Genotyping

DNA was extracted from blood using the Gentra Puregene kit (Qiagen, Maryland, USA), following the manufacturer’s protocol. Genotyping was performed using the Infinium Global Screening Array-24 BeadChip and Infinium PsychArray-24 BeadChip Illumina microarrays at the Institute of Psychiatry, Psychology & Neuroscience at King's College London. Genotyping quality control was carried out as recommended by Marees et al.^18^ Briefly, we removed markers with minor allele frequency (MAF) less than 0.01, missingness of SNVs higher than 0.1, and SNVs that deviated from Hardy-Weinberg equilibrium (p < 1 × 10^-6^). All parameters and cutoff points are listed in Supplementary Table S1.

Genotyping imputation was performed using the TOPMed Imputation Server according to the platform’s instructions. We then merged the imputed files (from each genotyping batch/chip) with PLINK version 1.9 software. To obtain independent (uncorrelated) SNVs, we removed the variants in linkage disequilibrium (r^2^ > 0.8).

### Selection of genomic regions

We selected 62 candidate genes (Supplementary Table S2) and their SNVs based on three criteria: 1) SNVs located in the gene body that participate in the pharmacodynamics (n=39) or pharmacokinetics (n=4) of risperidone as described in DrugBank (version 5.1.5) and the Drug Gene Interaction Database; 2) SNVs located in genes which exhibit differential expression after risperidone treatment (n=19), found in this FEP cohort in previous studies^19^-^22^; and 3) SNVs that regulate expression of the 62 selected candidate genes (cis-eQTLs) in brain tissue (amygdala, anterior cingulate cortex, caudate nucleus, frontal cortex, hippocampus, hypothalamus, nucleus accumbens, putamen, and substantia nigra), whole blood, or liver, obtained from the Genotype-Tissue Expression (version 8) portal, which might be located outside the gene boundaries (possibly not included in previous approaches). We used these criteria as we aimed to comprehensively capture genetic variations associated with response to risperidone. Quality control and extraction of SNVs were performed in PLINK software.

### Statistical analysis

Statistical tests were performed to compare responders and nonresponders with regard to continuous (Mann-Whitney *U* test) or categorical variables (Pearson’s chi-square test). Statistical significance was accepted at an α level < 0.05. Analyses were adjusted for multiple comparisons with Bonferroni correction for 11 comparisons (10 clinical variables and treatment time) and performed using R (version 3.6.1).

### Supervised machine learning

Supervised machine learning analyses were performed using the mlr package (version 2.19.0) in R software. We used three supervised classification algorithms: 1) support vector machine (SVM) using the radial basis function (RBF) kernel; 2) k-nearest neighbors (kNN); and 3) random forests (RF), all of which are often used in pharmacogenetic models.

### Prediction models

Each algorithm was used to create clinical, genetic, and hybrid models of response to risperidone treatment. The clinical models were designed using the following predictors: baseline CGI and GAF scores, sociodemographic data (age, biological sex, and household income), substance use (tobacco and cannabis) categorized as a dichotomous variable (patients who used substances in the past or never used them vs. patients who were currently using substances), risk factors (history of migration and psychosis in the family), and DUP.

The genetic models included all SNVs present in the regions of interest as predictor variables. Hybrid models were composed of two independent models: i) one containing all SNVs and all clinical variables; and ii) one containing clinical and genetic variables with feature selection (described below). SNV genotypes were categorized as 0 for homozygous allele 1, 1 for heterozygous, and 2 for homozygous allele 2. The statistical models could not be adjusted for principal components or any other procedure that considers the influence of genetic ancestry on prediction, since the method does not allow this type of adjustment.

### Model construction: training and testing

We used the five-fold nested cross-validation resampling method to partition the sample into training and testing subsets and avoid data leakage and overfitting. Stratified sampling was used to ensure that each fold of the cross-validation maintains the same proportion of each class as in the original dataset. The training sample was used to tune the hyperparameters (defined by the best performance in terms of balanced accuracy) of the SVM, kNN, and RF algorithms and was used as a test subsample in all the models. Details of the hyperparameter values are shown in Supplementary Table S3. For the hybrid model, an additional step of feature selection was applied with the “importance” method (randomForest R package), to increase the performance of the algorithms used in this study. Feature selection was applied along with hyperparameter tuning. The best model was chosen by selecting the highest performance observed for the average balanced accuracy. Precision, sensitivity, specificity, and area under the receiver operator characteristic curve (AUC-ROC) were also measured. Further, 95%CIs derived from five-fold cross-validation were calculated for all performance measures. The pipeline code is provided in the Supplementary Material S1.

### Ethics statement

All patients or their family members signed informed consent forms. The study protocol was approved by the National Research Ethics Committee (CONEP-CAAE 48242015.9.0000.5505).

## Results

### Performance of clinical models

The patients’ diagnoses are presented in Supplementary Table S4. The sample size varied according to the combination of available data. The number of predictors and individuals per model also varied, owing to the sensitivity of the resource selection to the algorithm used. The DUP was significantly different among responders (n=72) and nonresponders (n=69), even after correcting for multiple comparisons (Mann-Whitney *U*; adjusted p-value = 0.002), with a four-fold higher median value obtained for the nonresponder group. Sociodemographic variables, baseline psychiatric scale scores, risk factors, and use of cannabis and tobacco did not differ between the groups; in the additional analysis, the median duration of risperidone treatment likewise did not (Table 1).

**Figure 1:**
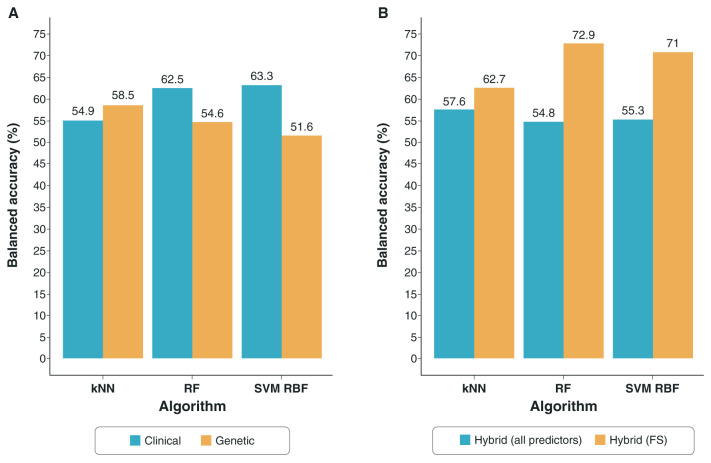
Balanced accuracy of prediction models. A) Clinical models (10 clinical predictors: (biological sex, migration, family psychosis history, cannabis, tobacco, Clinical Global Impression-Severity [CGI] score, Global Assessment of Functioning [GAF] score, duration of untreated psychosis [DUP], age, and household income) with 88 individuals; genetic models (1,265 predictor single-nucleotide variants [SNVs]) with 137 individuals. B) Hybrid models: without feature selection (10 clinical predictors and 1,265 predictor SNVs with 88 individuals), with feature selection (sample size varied depending on the combination of predictor variables available among the subjects). FS = feature selection; kNN = k-nearest neighbors; RF = random forests; SVM RBF = support vector machine using the radial basis function.

The clinical model with the best performance (highest balanced accuracy) was the one that used the SVM RBF algorithm and obtained (*C* hyperparameter: 10; *gamma* hyperparameter: 0.1), on average, a balanced accuracy of 63.3% (CI 0.46-0.69), precision of 74.1% (CI 0.44-0.65), sensitivity of 52.5% (CI 0.19-0.91), specificity of 74% (CI 0.25-0.95), and AUC-ROC of 61.6% (CI 0.51-0.73). Its high specificity shows its effectiveness in the prediction of nonresponse, while the low sensitivity shows that the model is weak in identifying responders. The average balanced accuracy values of the models and algorithms are shown in Figure 1A. Details on the performance measures are presented in Table 2 and Supplementary Figure S1A.

### Performance of genetic models

After quality control and imputation of the genomic data, 8,911,401 SNVs and 137 patients were used for further analysis (two outliers in heterozygosity and two individuals whose genotyping failed were excluded). Of these, 4,233 SNVs were extracted from the regions of interest, which may or may not be located within the open reading frame (ORF) of the genes, and were filtered by linkage disequilibrium. Due to the low MAF of some SNVs, some genotypes were absent in the training or test sample in the cross-validation iterations (a minimum of one genotype per group was required), which resulted in the exclusion of these variants. The final set comprised 1,265 SNVs that were included in the genetic models. The best genetic model that included all SNVs still performed poorly, with the kNN algorithm (*K* hyperparameter: 19) showing a balanced accuracy of 58.5% (CI 0.41-0.76) (Figure 1A), precision of 62.2% (CI 0.44-0.81), sensitivity of 48.6% (CI 0.30-0.67), specificity of 68.5% (CI 0.47-0.90), and AUC of 61.7% (CI 0.46-0.78). Details of the accuracy values are shown in Table 2. The ROC curve is shown in Supplementary Figure S1B.

### Performance of hybrid models

Hybrid models were tested with feature selection (feature selection hybrid models), prioritizing the importance of clinical and genetic variables for the models, and without feature selection (hybrid models). The hybrid model composed of a combination of 10 clinical and genetic variables (1,265 SNVs) showed the worst performance among all constructed models (hybrid, clinical, and genetic). The highest balanced accuracy (57.6%, CI 0.42-0.73) was obtained by the kNN algorithm (Figure 1B) (*K* hyperparameter: 15), with a precision of 55.6% (CI 0.31-0.80), sensitivity of 40% (CI 0.14-0.66), specificity of 75.1% (CI 0.57-0.94), and AUC-ROC of 61.8% (CI 0.44-0.79) (Table 2 and Supplementary Figure S1C).

**Figure 2:**
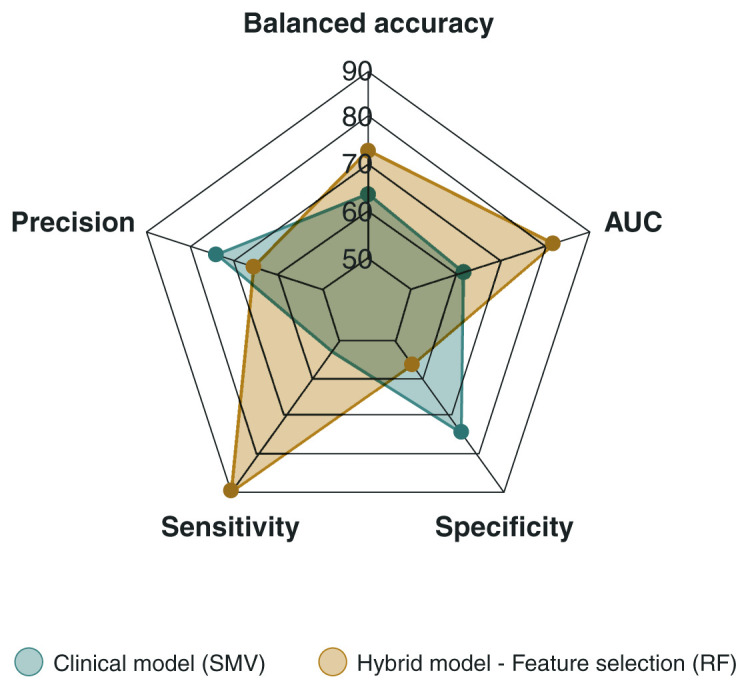
Radar graph of performance measures of the best clinical and hybrid models. AUC = area under the receiver operator characteristic (ROC) curve; Clinical model (SVM) = support vector machine algorithm; Hybrid model - Feature selection (RF) = model with random forests algorithm and feature selection.

Application of the RF importance filter method contributed to a remarkable increase in the performance of the prediction models, surpassing previous predictions and achieving the highest balanced accuracy. This result was obtained using the RF algorithm (*ntree* hyperparameter: 500; *mtry* hyperparameter:100), showing a balanced accuracy of 72.9% (CI 0.62-0.84) (Figure 1B). Furthermore, the sensitivity (89.6%; CI 0.80-0.99) and AUC-ROC (81.8%; CI 0.67-0.97) of this model were the highest among all predictions, even when compared to the clinical model. However, its performance was not satisfactory in terms of precision (65.7%; CI 0.57-0.74) and specificity (56.2%; CI 0.36-0.76) (Figure 2), indicating that the hybrid model with feature selection has a high capacity for identifying responders but low efficiency in classifying nonresponders. The performance measures of the other algorithms are presented in Table 2 and Supplementary Figure S1D.

The most important predictors for the hybrid model prioritized by feature selection (importance) were DUP, CGI, age, cannabis use (clinical), and 406 SNVs (genetics). Details of the selected SNVs are provided in Supplementary Table S5. The number of clinical and genetic predictors selected by other algorithms, as well as details of the clinical variables chosen by feature selection, are presented in Supplementary Table S6. Most of the selected SNVs were present in intron regions (69.2%), six were missense variants (rs12793222, rs337285, rs4680, rs2228314, rs745142, and rs1805123), four were synonymous variants (rs2070116, rs1052706, rs8040868, and rs2229422), and three were located in the regulatory regions of the genome (rs9787309, rs10885090, and rs4767469), as described in Supplementary Figure S2.

Approximately 201 SNVs were eQTLs for 31 candidate genes and were associated with gene expression alterations, mostly in whole blood (n=118 SNVs), brain (n=78 SNVs), and liver (n=5 SNVs). *CYP2D6* was the gene whose expression was most strongly influenced by the model variants. Four SNVs showed altered expression in the brain and 34 SNVs in whole blood (Supplementary Figure S3).

## Discussion

Herein, we used clinical and genetic data – separately and together, i.e., in hybrid models – to predict patients’ response to antipsychotic therapy using a supervised machine learning approach. We examined a cohort of antipsychotic-naive patients who were treated exclusively with risperidone after experiencing FEP.

### Clinical models

Clinical models were created using functional measures, sociodemographic information, DUP, substance use, and risk factor data. These variables were not statistically different between the responder and nonresponder groups, except for the DUP, which was longer for nonresponders. Longer DUP has been associated with increased severity of negative and positive symptoms and decline in global functioning.^5^,^23^ Although the DUP in our sample was shorter than the previously reported average of 2 years,^24^ this variable showed great potential for predicting treatment response. Our best clinical prediction model (balanced accuracy: 63.3%) stood out for its ability to effectively predict nonresponders using the SVM algorithm; however, it showed poor performance in predicting responders. Some studies have used clinical/sociodemographic data in machine learning approaches to predict response to lithium (balanced accuracy: 77%)^25^ and antidepressants (balanced accuracy: 88%) in bipolar disorder.^26^ Regarding response to antipsychotics, Ambrosen et al.^27^ obtained a balanced accuracy of 50-50.3% for response rating, while Koutsouleris et al.^28^ obtained a balanced accuracy of 73.8-75%. Cao’s model achieved excellent performance (balanced accuracy: 82.5%); his model was able to predict a PANSS response rate of 30% using algorithms belonging to the SVM family with MRI data in a treated FEP cohort However, the sample size was small.^29^ Compared to current models, which used different sample sizes, methods, PANSS scores, and variables from those in our study, our model performed moderately well.

### Genetic models

Models constructed strictly with genetic variants exhibited lower performance. To our knowledge, no study has exclusively used genetic variants to predict response to antipsychotic drugs using machine learning methods. Most studies have applied these methods to predict schizophrenia cases and controls^30^ or have combined genetic data with other variables in hybrid models, as we discuss in the next section.

### Hybrid models

Our unfiltered hybrid model presented a low balanced accuracy, close to the margins of the metrics obtained with the above models, indicating that the genetic variants reduced the performance of the clinical model. To exclude noisy and redundant variables that disturb the model, we used feature selection, which was able to select the most important predictors and substantially increase accuracy.

Our hybrid model with feature selection obtained a balanced accuracy of 72.9%, largely because of the presence of DUP, which has gained visibility within the field of early treatment intervention. Considering that the first years of the disease have a greater impact on the progression of psychosis than the following years,^31^ reducing DUP may help improve prognosis. Another modifiable factor selected was the use of cannabis, which is associated with relapse of psychosis, increased risk of hospital admission, and decline in the response to antipsychotic treatment in the post-FEP period.^32^ In addition, patients who use cannabis are at greater risk of nonadherence to pharmacotherapy.^33^ This scenario is of importance from an interventionist’s perspective, as it allows one to design guidance strategies for patients.

The occurrence of psychotic symptoms early in an individual’s life is related to an increased genetic predisposition, poor prognosis, and symptom severity,^34^ as well as poor treatment response.^5^ In this study, this factor was relevant in predicting the response to risperidone, as were measures from the CGI scale, which assesses disease severity and is used to measure the response to antipsychotics.^35^ Although age at onset and CGI score at the time of FEP are not modifiable, these variables – which constitute the best prediction model – require observation in clinical management, as they may be risk factors for poor treatment response.

Most genetic variants selected in the hybrid model were in the intron regions, and half of the SNVs could alter gene expression, mainly in whole blood. Most eQTLs altered the expression of the *CYP2D6* gene (whole blood and brain), probably due to the larger number of eQTLs associated with this gene than with other candidate genes in the present study. This gene is highly polymorphic, and the protein it encodes is the main enzyme that metabolizes risperidone in the liver; however, the model did not include any eQTLs known to alter its expression in hepatic tissue.

Among the existing literature that applied machine learning methods using hybrid models, we highlight two studies with different performance outcomes than ours. Lee et al.^36^ utilized clinical profiles and sociodemographic data (53 variables), along with candidate SNVs, to predict the response to various antipsychotics, including risperidone, in non-drug-naive individuals using a machine learning algorithm. The SNVs were selected from loci associated with schizophrenia in two genome-wide association studies; their accuracies were 60% (13 SNVs) and 57% (25 SNVs) for predicting the response to risperidone. These values were lower than those reported for our hybrid model. Lee et al.^36^ did not select genetic variants associated with clinical data and did not use imputed genetic data, potentially missing important SNVs for their model. In contrast, our model used a cohort of drug-naive FEP patients, performed genomic data imputation, used SNVs related to the response to risperidone treatment, and filtered the most important variables, all of which contributed to enhanced model performance. In a recent study, Guo et al.^37^ combined clinical data (PANSS score at baseline, antipsychotic drug, sex, and age), polygenic risk score, genetic risk score, and proxy DNA methylation, achieving high AUCs (0.874 in the discovery cohort and 0.851 in an external validation cohort) in one of the largest multi-omics studies of treatment response to date. Although their performance was superior to ours, it is important to note that their study population was Chinese and not restricted to FEP patients. These differences highlight the novelty and potential clinical utility of our approach in a Brazilian FEP cohort.

This study has several limitations. First, the sample size was small due to the challenges in accessing and monitoring the drug-naive FEP cohort with acute psychosis. Specifically, this cohort has been followed since 2011, and only 57% of individuals assessed at screening were included, with only 65% assessed after 2 months. Second, genetic ancestry may introduce bias in the models, particularly given the highly admixed nature of the Brazilian population, limiting the generalizability of our findings to other populations. Additional studies in diverse populations are needed. Third, we focused on common SNVs, which limits the genetic scope of our study. Incorporating rare variants or DNA methylation data could improve predictive performance. Additionally, investigating responses to other antipsychotic drugs over longer follow-up periods is crucial for enhancing the generalizability of our findings and understanding the relevance of key variables in different contexts. Lastly, using injectable antipsychotic drugs would help determine if treatment adherence affected our results. While our hybrid model shows promise, larger and more diverse cohorts are essential to enhance its generalizability.

In conclusion, a hybrid model combining clinical and genetic factors with feature selection can achieve a clinically meaningful performance in predicting the response to risperidone treatment. When analyzed in isolation, the clinical model showed greater performance than strictly genetic models (SNVs). In addition, modifiable clinical predictors, risk factors for mental disorders, and patients’ psychiatric data, together with SNVs capable of altering the expression of genes involved in antipsychotic metabolism, can be used jointly to improve response prediction. Considering that most studies using machine learning methods to predict antipsychotic response have focused on neuroimaging biomarkers,^38^ this study is the first to investigate this outcome in the Brazilian population using genetic variants. Future analyses in larger and more diverse populations, incorporating hybrid models, are necessary to explore the interactions among these predictors, identify additional factors that contribute to model performance, and advance precision medicine.

## Disclosure

RAB declares personal fees and non-financial support from Janssen and Ache Laboratórios Farmacêuticos; grants and personal fees from Roche, all outside the submitted work. AG declares personal fees and non-financial support from Janssen, Daiichi Sankyo, Lundbeck, Teva, Cristalia, and Ache Laboratórios Farmacêuticos, outside the submitted work. CN declares personal fees and non-financial support from Janssen, Daiichi Sankyo, Lundbeck, Teva, and Ache Laboratórios Farmacêuticos, outside the submitted work. The other authors report no conflicts of interest.

## Figures and Tables

**Table 1:** Statistical analysis of clinical variables

Variables	Ns	Responders		Ns	Nonresponders		Statistics	p-value	Adjusted p-value
Biological sex	72	M	46 (63.9)	69	M	44 (63.8)	χ^2^ = 0.000	1.000	> 0.05
		F	26 (36.1)		F	25 (36.2)			
Migration	67	N	44 (65.7)	65	N	34 (52.3)	χ^2^ = 1.916	0.166	> 0.05
		Y	23 (34.3)		Y	31 (47.7)			
Family history of psychosis	69	N	27 (39.1)	62	N	35 (56.5)	χ^2^ = 3.266	0.071	> 0.05
		Y	42 (60.9)		Y	27 (43.5)			
Cannabis use	59	N	37 (62.7)	61	N	51 (83.6)	χ^2^ = 5.670	0.017	> 0.05
		Y	22 (37.3)		Y	10 (16.4)			
Smoking	67	N	55 (82.1)	67	N	52 (77.6)	χ^2^ = 0.186	0.667	> 0.05
		Y	12 (17.9)		Y	15 (22.4)			
CGI (md [IQR])	67	5 (2.00)		65	5 (1.00)		U = 2,089.0	0.668	> 0.05
GAF (md [IQR])	69	30 (15.00)		66	30 (10.00)		U = 2,327.5	0.823	> 0.05
DUP (md [IQR])	67	19 (61.50)		65	77 (180.00)		U = 3,000.5	< 0.001	0.002*
Age (md [IQR])	72	22 (9.25)		69	23 (10.00)		U = 2,782.5	0.218	> 0.05
Household income in USD (md [IQR])	67	2,500 (1,900)		64	2,300 (2,000)		U = 2,064.5	0.715	> 0.05
Treatment duration (md [IQR])	72	68 (16.50)		69	65 (18.00)		U = 2,267.5	0.373	> 0.05

Data presented as n (%), unless otherwise specified.

Adjusted p-value corrected for 11 comparisons.

Household income converted from Brazilian reals to United States dollars using the mean August 2022 exchange rate (1.00 USD = 5.14 BRL).

CGI = Clinical Global Impression-Severity; DUP = duration of untreated psychosis; F = female; GAF = Global Assessment of Functioning Scale; IQR = interquartile range; M = male; md = median; N = no; Ns = sample size; Y = yes.

* p < 0.05.

**Table 2:** Model performance

Model/algorithm	Balanced accuracy, % (CI)	Precision, % (CI)	Sensitivity, % (CI)	Specificity, % (CI)	AUC, % (CI)
Clinical					
SVM RBF	63.3 (0.46-0.69)	74.1 (0.44-0.65)	52.5 (0.19-0.91)	74.0(0.25-0.95)	61.6 (0.51-0.73)
kNN	54.9 (0.49-0.82)	50.6 (0.43-0.81)	47.5 (0.39-0.91)	62.2(0.46-0.87)	60.5 (0.58-0.84)
RF	62.5 (0.48-0.73)	59.7 (0.45-0.70)	55.0 (0.25-0.75)	70.0 (0.59-0.83)	68.2 (0.48-0.77)
Genetic					
SVM RBF	51.6 (0.45-0.59)	53.5 (0.44-0.63)	71.4 (0.36-1.07)	31.8 (0.09-0.72)	56.7 (0.46-0.68)
kNN	58.5 (0.41-0.76)	62.2 (0.44-0.81)	48.6 (0.30-0.67)	68.5 (0.47-0.90)	61.7 (0.46-0.78)
RF	54.6 (0.42-0.68)	55.3 (0.44-0.67)	62.9 (0.48-0.77)	46.4 (0.30-0.63)	57.7 (0.45-0.70)
Hybrid					
SVM RBF	55.3 (0.48-0.63)	42.5 (0.12-0.73)	37.5 (0.01-0.74)	73.1 (0.47-0.99)	55.0 (0.51-0.59)
kNN	57.6 (0.42-0.73)	55.6 (0.31-0.80)	40.0 (0.14-0.66)	75.1 (0.57-0.94)	61.8 (0.44-0.79)
RF	54.8 (0.46-0.64)	52.0 (0.36-0.68)	32.5 (0.15-0.50)	77.1 (0.72-0.82)	51.6 (0.38-0.65)
Hybrid (FS)					
SVM RBF	71.0 (0.57-0.85)	68.1 (0.56-0.80)	72.9 (0.54-0.92)	69.1 (0.59-0.79)	75.6 (0.63-0.88)
kNN	62.7 (0.52-0.74)	60.2 (0.46-0.75)	75.3 (0.64-0.87)	50.2 (0.25-0.75)	75.8 (0.69-0.82)
RF	72.9 (0.62-0.84)	65.7 (0.57-0.74)	89.6 (0.80-0.99)	56.2 (0.36-0.76)	81.8 (0.67-0.97)

95%CI in 5-fold cross-validation.

AUC = area under the receiver operating characteristic (ROC) curve; FS = feature selection; kNN = k-nearest neighbors; RF = random forests; SVM RBF = support vector machine using the radial basis function.
