# Supplementary material for: A hybrid model for predicting response to risperidone after first-episode psychosis
Source: Braz J Psychiatry. 2025 Jan 22;47:e20243608. doi: 10.47626/1516-4446-2024-3608 (PMC12679696; doi:10.47626/1516-4446-2024-3608)
Supplement: Supplementary file 1 [file bjp-47-e20243608-suppl1.pdf]

http://doi.org/10.47626/1516-4446-2024-3608


**Supplementary Table S1** Genotyping quality control parameters and cut-offs

| Parameter | Cut-off points |
|---|---|
| MAF | 1% |
| Missingness of SNVs | 10% |
| HWE | $1\times10^{-6}$ |
| Missingness of individuals | 10% |
| Heterozygosity | ±3 SD |
| IBD | 0.125 |
| Sex discrepancy | 0.8 (male) or 0.2 (female) |

HWE = Hardy-Weinberg equilibrium; IBD = identity-by-descent; MAF = minor allele frequency; SNVs = single-nucleotide variants.
SNVs with MAF < 1%, missingness of SNVs > 10%, and SNVs that deviated from HWE ($p < 1\times10^{-6}$) were removed. Samples were removed in the following situations: individual missingness > 10%, with deviation ±3 from the heterozygosity rate mean; IBD > 0.125 or individuals discrepant from the X chromosome homozygosity estimate (> 0.8 for men and < 0.2 for women). Additionally, pseudo-autosomal regions, mitochondrial genomes, sex chromosomes, and indels were removed.

**Supplementary Table S2** Candidate genes

| Gene class | Gene |
|---|---|
| Pharmacokinetics | *ABCB1*,† *CYP2D6*,‡ *CYP3A4*,‡ *CYP1A1*§ |
| Pharmacodynamics | *HTR2A*,‡ *DRD2*,‡ *ADRA1A*,‡ *DRD3*,‡ *DRD4*,‡ *ADRA1B*,‡ *HTR1D*,‡ *HRH1*,‡ *ADRA2A*,‡ *ADRA2B*,‡ *ADRA2C*,‡ *HTR1A*,‡ *DRD1*,‡ *HTR7*,† *HTR1B*,§ *HTR1E*,§ *HTR1F*,§ *HTR6*,§ *SERPINA3*,§ *TNF*,§ *COMT*,§ *NTF3*,§ *E2F3*,§ *SLC6A4*,§ *GRM3*,§ *ZBTB42*,§ *E2F2*,§ *ANKK1*,§ *NTS*,§ *AKT1*,§ *FASN*,§ *LEP*,§ *SLC1A1*,§ *KCNH2*,§ *RGS4*,§ *GHRL*,§ *FAAH*,§ *ARVCF*,§ *ADRB2*§ |
| Differentially expressed after risperidone treatment | *MBP*, *NDEL1*, *GCH1*, *GABRR2*, *CHRNA3*, *C1orf128*, *ADIPOR1*, *GMPR*, *SNCA*, *WDR40A*, *TESC*, *MAP2K3*, *ST6GALNAC4*, *DCAF6*, *HAGH*, *BCL2L1*, *FBXO7*, *FAM104A*, *DMTN* |

† Genes exclusively reported in DrugBank.
‡ Genes reported in DrugBank and DGIdb. Access date: October 28, 2019.
§ Genes exclusively reported in the DGIdb. Access date: October 28, 2019.

**Supplementary Table S3** Hyperparameters adjusted for each algorithm and their respective values

| | Set of analyzed values | | | |
|---|---|---|---|---|
| **Algorithm/ hyperparameters** | **Clinical model** | **Genetic model** | **Hybrid model (all predictors)** | **Hybrid model (feature selection)** |
| SVM | | | | |
| C† | - | - | 0.001; 0.01; 0.1; 1; 10 | - |
| Gamma‡ | - | - | 0.001; 0.01; 0.1; 1; 10 | - |
| kNN | | | | |
| K§ | - | - | 1:20 | - |
| RF | | | | |
| mtry‖ | - | - | 500; 600; 700; 800; 900; 1000 | - |
| ntree¶ | 1:10 | 100; 300; 600; 900; 1200 | 100; 300; 600; 900; 1200 | 100; 300; 600; 900 |

kNN = k-nearest neighbors; RF = random forests; SVM = support vector machine.
† Hyperparameter of hyperplane regularization, which penalizes erroneously classified observations by increasing the margin range.
‡ Analyzes the influence of close individual observations on the classification in the model.
§ Number of points predetermined by the algorithm to classify the observations according to the distance between them.
‖ Number of variables randomly selected for use at each node to divide the tree in the RF.
¶ Number of trees formed in each model of the RF.
Each hyperparameter value was analyzed using a grid search, an iterative method, standard of *mlr*, able to optimize the choice of values, test the combination of each of them, evaluate their average performance using a resampling method (nested cross-validation), and select the hyperparameter value that resulted in the best balanced accuracy.

http://doi.org/10.47626/1516-4446-2024-3608


## Supplementary Material S1

Pipeline coding in R used to conduct the Machine Learning models

```
## Load packages
library(mlr)
library(tidyverse)
library(kernlab)
library(kknn)
library(randomForest)
library(caTools)

##### Clinical data preparation #####
# a) Importing the file
data <- read.table("PEP_DC_DSD.txt", header = TRUE, sep= "\t", row.names= "ID")

# b) Creating a column containing the percentage of treatment response
data$Complete_Response <- ((data$Pansstot_1 - 30) - (data$Pansstot_2 - 30))*100/(data$Pansstot_1-30)

# c) Creating response groups
data$Responder <- ifelse(data$Complete_Response > 50, 1, 2)

# d) Transforming the outcome variable (numeric) into a factor (necessary for classification)
data$Responder <- as.factor(data$Responder)

data$Gender <- as.factor(data$Gender)
data$Migration <- as.factor(data$Migration)
data$Family_Psych_History <- as.factor(data$Family_Psych_History)
data$Marijuana <- as.factor(data$Marijuana)
data$Alcohol <- as.factor(data$Alcohol)
data$Cigarette <- as.factor(data$Cigarette)

# e) Creating a dataset with the final variables for analysis
data <- subset(data, select=c("CGI", # Baseline CGI total
                              "GAF", # Baseline GAF
                              "DUP", # DUP
                              "Age", # Age
                              "Family_Income", # Family income
                              "Gender", # Gender
                              "Migration", # Migration
                              "Family_Psych_History", # Family history of psychosis
                              "Marijuana", # Marijuana use
                              "Cigarette", # Cigarette use
                              "Responder")) # Cut-off 50%

# f) Normalizing data by Z-score (Mainly for SVM and KNN)
data$CGI <- as.numeric(data$CGI)
data$GAF <- as.numeric(data$GAF)
data$DUP <- as.numeric(data$DUP)
data$Age <- as.numeric(data$Age)
data$Family_Income <- as.numeric(data$Family_Income)

data <- as.data.frame(scale(data[1:5]))

########## Machine Learning Clinical Models ##########
# Create a classification task
class.task = makeClassifTask(id = deparse(substitute(data)), data = data, target = "Responder")

# Performance measures
performance_measures <- list(acc, bac, ppv, tpr, tnr, auc)

########## SVM ##########
# I) Training the model

method.SVM <- makeLearner("classif.ksvm", predict.type = "prob")

# Define a 5-fold cross-validation resampling strategy
```

http://doi.org/10.47626/1516-4446-2024-3608


```
est_amost5CV <- makeResampleDesc("CV", iters = 5, predict = "both", stratify = T)

# Define SVM hyperparameters to be tuned
svm_param <- makeParamSet(
  makeDiscreteParam("C", values = c(0.001, 0.01, 0.1, 1, 10)),
  makeDiscreteParam("sigma", values = c(0.001, 0.01, 0.1, 1, 10))
)

# Create a grid search control to explore all combinations of parameters
svm_busca <- makeTuneControlGrid()

# Set a seed for reproducibility
set.seed(1000)

# Define additional performance measures for the training set
accTrainMean = setAggregation(acc, train.mean)
bacTrainMean = setAggregation(bac, train.mean)
ppvTrainMean = setAggregation(ppv, train.mean)
tprTrainMean = setAggregation(tpr, train.mean)
tnrTrainMean = setAggregation(tnr, train.mean)
aucTrainMean = setAggregation(auc, train.mean)

# Perform hyperparameter tuning with cross-validation
tunning_svm <- tuneParams(
  learner = method.SVM,
  task = class.task,
  resampling = est_amost5CV,
  par.set = svm_param,
  control = svm_busca,
  measures = bac # Use balanced accuracy as the performance measure
)

# Set the best hyperparameters found during tuning
method.SVM.adjusted <- setHyperPars(method.SVM, par.vals = tunning_svm$x)

# Calculate the performance
r_ajust = resample(method.SVM.adjusted, class.task, rdesc_5CV, measures = list(acc, accTrainMean,
                                                 bac, bacTrainMean,
                                                 ppv, ppvTrainMean,
                                                 tpr, tprTrainMean,
                                                 tnr, tnrTrainMean,
                                                 auc, aucTrainMean))

# Output of the performance measures
r_ajust$aggr

########## KNN ##########
# I) Training the model

method.KNN <- makeLearner("classif.kknn", predict.type = "prob")

# Define KNN hyperparameters to be tuned
KNN_param <- makeParamSet(makeDiscreteParam("k", values = c(1:20)))

# Create a grid search control to explore all combinations of parameters
KNN_busca <- makeTuneControlGrid()

# Set a seed for reproducibility
set.seed(1000)

# Perform hyperparameter tuning with cross-validation
tunning_KNN <- tuneParams(
  learner = method.KNN,
  task = class.task,
  resampling = est_amost5CV,
  par.set = KNN_param,
  control = KNN_busca,
  measures = bac # Use balanced accuracy as the performance measure
```

```
)

# Set the best hyperparameters found during tuning
method.KNN.adjusted <- setHyperPars(method.KNN, par.vals = tunning_KNN$x)

# Calculate the performance
r_ajust = resample(method.KNN.adjusted, class.task, rdesc_5CV, measures = list(acc, accTrainMean,
                                                         bac, bacTrainMean,
                                                         ppv, ppvTrainMean,
                                                         tpr, tprTrainMean,
                                                         tnr, tnrTrainMean,
                                                         auc, aucTrainMean))

# Output of the performance measures
r_ajust$aggr

########## Random Forest ##########
# I) Training the model

method.RF <- makeLearner("classif.randomForest", predict.type = "prob", par.vals = list(importance=T))

# Define RF hyperparameters to be tuned
RF_param <- makeParamSet(
  makeDiscreteParam("ntree", values = c(500,600,700,800,900,1000)),
  makeDiscreteParam("mtry", values = c(1,2,3,4,5,6,7,8,9,10))
)

# Create a grid search control to explore all combinations of parameters
RF_busca <- makeTuneControlGrid()

# Set a seed for reproducibility
set.seed(1000)

# Perform hyperparameter tuning with cross-validation
tunning_RF <- tuneParams(
  learner = method.RF,
  task = class.task,
  resampling = est_amost5CV,
  par.set = RF_param,
  control = RF_busca,
  measures = bac # Use balanced accuracy as the performance measure
)

# Set the best hyperparameters found during tuning
method.RF.adjusted <- setHyperPars(method.RF, par.vals = tunning_RF$x)

# Calculate the performance
r_ajust = resample(method.RF.adjusted, class.task, rdesc_5CV, measures = list(acc, accTrainMean,
                                                         bac, bacTrainMean,
                                                         ppv, ppvTrainMean,
                                                         tpr, tprTrainMean,
                                                         tnr, tnrTrainMean,
                                                         auc, aucTrainMean))

# Output of the performance measures
r_ajust$aggr

##### Genetic data preparation #####

# a) Importing the VCF file
Genetic_Data <- read.table("vcf_table.txt", dec = ",")

# b) Replacing genotypes with categories: 0, 1, and 2
Genetic_Data <- apply(Genetic_Data, 2, function(y) gsub("0/0", "0", y))
Genetic_Data <- apply(Genetic_Data, 2, function(y) gsub("0/1", "1", y))
Genetic_Data <- apply(Genetic_Data, 2, function(y) gsub("1/1", "2", y))
Genetic_Data <- apply(Genetic_Data, 2, function(y) gsub("./.", "", y))
```

http://doi.org/10.47626/1516-4446-2024-3608
A hybrid model for predicting response to risperidone after first-episode psychosis – Costa GO et al.

```
# c) Transposing the file
Genetic_Data <- t(Genetic_Data)
Genetic_Data <- as.data.frame(Genetic_Data)
#Verify if the Genetic_Data is in the same order as the clinical data and then include the outcome in the Genetic Data
Genetic_Data$Responder<- data$Responder

########## Machine Learning Genetic Models ##########
# perform all the same models using the "Genetic_Data" instead of "data"
# modify "# "mtry" parametrs to values = c(10,50,100,150,200,250,300)

# d) Merging genetic data with each outcome (clinical data) individually

########## Machine Learning Hybrid Models ##########
# Merging clinical and genetic files

Merge_Clinical_Genetic <- join_all(list(Genetic_Data, data), by="ID", type = "left")
row.names(Merge_Clinical_Genetic) <- Merge_Clinical_Genetic$ID

class.task = makeClassifTask(id = deparse(substitute(data)), data = Merge_Clinical_Genetic, target = "Responder")

########## SVM ##########
# I) Training the model

method.SVM <- makeLearner("classif.ksvm", predict.type = "prob")
lrn = makeFilterWrapper(learner = method.SVM,
                        fw.method = "randomForest_importance")

x0 = 0.05
seq <- seq(x0, 1, 0.05)

search_space = makeParamSet(makeDiscreteParam("fw.perc", values = seq),
                            makeDiscreteParam("C", values = c(0.001,0.01,0.1,1,10)),
                            makeDiscreteParam("sigma", values = c(0.001,0.01,0.1,1,10)))

ctrl = makeTuneControlGrid()

# Define a 5-fold cross-validation resampling strategy
est_amost5CV <- makeResampleDesc("CV", iters = 5, predict = "both", stratify = T)

set.seed(1000)
res <- tuneParams(learner = lrn,
                  task = class.task,
                  resampling = est_amost5CV,
                  par.set = search_space,
                  control = ctrl,
                  measures= bac)

lrn_ajust <- setHyperPars(learner = lrn,
                          par.vals = res$x)

set.seed(1000)

# Calculate the performance
r_ajust = resample(lrn_ajust, class.task, est_amost5CV, measures = list(acc, accTrainMean,
                                                                        bac, bacTrainMean,
                                                                        ppv, ppvTrainMean,
                                                                        tpr, tprTrainMean,
                                                                        tnr, tnrTrainMean,
                                                                        auc, aucTrainMean))

# Output of the performance measures
r_ajust$aggr

########## KNN ##########
# I) Training the model

method.KNN <- makeLearner("classif.kknn", predict.type = "prob")
```

```
lrn = makeFilterWrapper(learner = method.KNN,
                        fw.method = "randomForest_importance")

x0 = 0.05
seq <- seq(x0, 1, 0.05)

search_space = makeParamSet(makeDiscreteParam("fw.perc", values = seq),
                            makeDiscreteParam("k", values = c(1:20)))
ctrl = makeTuneControlGrid()

set.seed(1000)
res <- tuneParams(learner = lrn,
                  task = class.task,
                  resampling = est_amost5CV,
                  par.set = search_space,
                  control = ctrl,
                  measures= bac)

lrn_ajust <- setHyperPars(learner = lrn,
                          par.vals = res$x)

set.seed(1000)

# Calculate the performance
r_ajust = resample(lrn_ajust, class.task, est_amost5CV, measures = list(acc, accTrainMean,
                                                                         bac, bacTrainMean,
                                                                         ppv, ppvTrainMean,
                                                                         tpr, tprTrainMean,
                                                                         tnr, tnrTrainMean,
                                                                         auc, aucTrainMean))

# Output of the performance measures
r_ajust$aggr
########## Random Forest ##########
# I) Training the model

method.RF <- makeLearner("classif.randomForest", predict.type = "prob", par.vals = list(importance=T))

lrn = makeFilterWrapper(learner = method.KNN,
                        fw.method = "randomForest_importance")

x0 = 0.05
seq <- seq(x0, 1, 0.05)

search_space = makeParamSet(makeDiscreteParam("fw.perc", values = seq),
                            makeDiscreteParam("ntree", values = c(500,600,700,800,900,1000)),
                            makeDiscreteParam("mtry", values = c(10,50,100,150,200)))
ctrl = makeTuneControlGrid()

set.seed(1000)
res <- tuneParams(learner = lrn,
                  task = class.task,
                  resampling = est_amost5CV,
                  par.set = search_space,
                  control = ctrl,
                  measures= bac)

lrn_ajust <- setHyperPars(learner = lrn,
                          par.vals = res$x)

set.seed(1000)

# Calculate the performance
r_ajust = resample(lrn_ajust, class.task, est_amost5CV, measures = list(acc, accTrainMean,
                                                                         bac, bacTrainMean,
                                                                         ppv, ppvTrainMean,
                                                                         tpr, tprTrainMean,
                                                                         tnr, tnrTrainMean,
```

```
                                                auc, aucTrainMean))

# Output of the performance measures
r_ajust$aggr

# Define RF hyperparameters to be tuned
RF_param <- makeParamSet(
  makeDiscreteParam("ntree", values = c(500,600,700,800,900,1000)),
  makeDiscreteParam("mtry", values = c(1,2,3,4,5,6,7,8,9,10))
)

# Create a grid search control to explore all combinations of parameters
RF_busca <- makeTuneControlGrid()

# Set a seed for reproducibility
set.seed(1000)

# Perform hyperparameter tuning with cross-validation
tunning_RF <- tuneParams(
  learner = method.RF,
  task = class.task,
  resampling = est_amost5CV,
  par.set = RF_param,
  control = RF_busca,
  measures = bac # Use balanced accuracy as the performance measure
)

# Set the best hyperparameters found during tuning
method.RF.adjusted <- setHyperPars(method.RF, par.vals = tunning_RF$x)


# Calculate the performance
r_ajust = resample(method.RF.adjusted, class.task, rdesc_5CV, measures = list(acc, accTrainMean,
                                                bac, bacTrainMean,
                                                ppv, ppvTrainMean,
                                                tpr, tprTrainMean,
                                                tnr, tnrTrainMean,
                                                auc, aucTrainMean))

# Output of the performance measures
r_ajust$aggr

#####ROC curve#####
roc.pred.teste <- generateThreshVsPerfData(list(RL = pred.model.RL.test,
                                        SVM = pred.model.SVM.adjusted.test,
                                        KNN = pred.modelo.KNN.adjusted.test,
                                        RF = pred.modelo.RF.adjusted.test),
                                     measures = list(fpr, tpr))
plotROCCurves(roc.pred.teste)
```

**Supplementary Table S4** Diagnoses of study participants (n=101)

| **Diagnosis** | **n (%)** |
|---|---|
| Schizophrenia spectrum | 56 (39.7) |
| Manic episode with psychotic symptoms | 21 (14.9) |
| Severe depressive episode with psychotic symptoms | 8 (5.7) |
| Other psychosis† | 56 (39.7) |

Follow-up: 73.7±29.0 days.
† Delusional disorder, brief psychotic disorder, substance-induced psychotic disorder, psychotic disorder not otherwise specified.

**Supplementary Figure S1** Receiver operator characteristic curves of prediction models

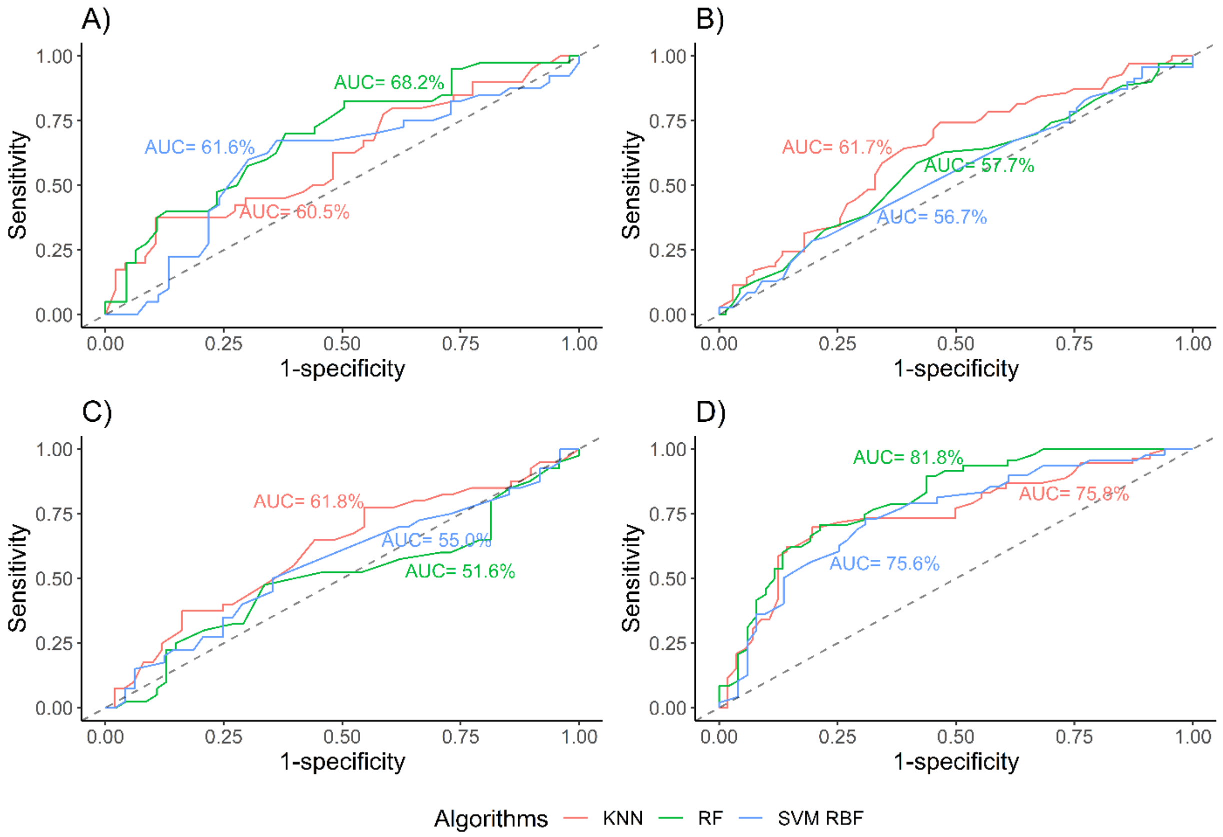


AUC = area under the curve; CGI = Clinical Global Impression-Severity; DUP = duration of untreated psychosis; GAF = Global Assessment of Functioning; kNN: k-nearest neighbors; RF: random forests; SNV = single-nucleotide variants; SVM RBF: support vector machine radial basis function.
(A) Clinical model containing 10 predictors (sex, migration, family psychosis history, cannabis, smoke, baseline CGI score, baseline GAF score, DUP, age, and household income). (B) genetic model containing 1,265 predictive SNVs. (C) hybrid model containing all clinical and genetic variables. (D) hybrid model with feature selection, containing the variables chosen by the feature selection method (CGI, DUP, age, cannabis use, and 406 SNVs).

**Supplementary Table S5** Annotations of the SNVs selected by importance in the hybrid model

| Chromosome | SNV ID | Position | Allele | Consequence | Gene | AGE | Tissue |
|---|---|---|---|---|---|---|---|
| 1 | rs2038027 | 23521550 | G | intron | *E2F2* | - | - |
| 1 | rs202266 | 167960545 | T | intron | *DCAF6* | - | - |
| 1 | rs7539542 | 202940846 | C | 3-prime UTR | *ADIPOR1* | - | - |
| 1 | rs1342387 | 202945228 | C | intron | *ADIPOR1* | - | - |
| 1 | rs10800887 | 202955808 | T | intron | *ADIPOR1* | - | - |
| 1 | rs10917481 | 19539821 | G | intron | *MICOS10* | *HTR6* | nucleus accumbens |
| 1 | rs34087508 | 19563837 | C | intron | *MICOS10* | *HTR6* | whole blood |
| 1 | rs1894254 | 19651031 | A | intron | *NBL1* | *HTR6* | whole blood |
| 1 | rs3087566 | 19657968 | T | downstream gene | *NBL1* | *HTR6* | whole blood |
| 1 | rs4912140 | 19674578 | G | intron | *HTR6* | *HTR6* | whole blood |
| 1 | rs6677765 | 19685970 | G | intron | *TMCO4* | *HTR6* | whole blood |
| 1 | rs3934191 | 19700079 | A | intron | *TMCO4* | *HTR6* | whole blood |
| 1 | rs7547898 | 19700265 | T | intron | *TMCO4* | *HTR6* | whole blood |
| 1 | rs10917330 | 22932151 | T | intergenic | - | *HTR1D* | cortex |
| 1 | rs722993 | 22940597 | G | intergenic | - | *HTR1D* | cortex |
| 1 | rs10799781 | 22987338 | T | intergenic | - | *HTR1D* | cortex |
| 1 | rs1208930 | 23105853 | T | intron | *LUZP1* | *HTR1D* | cortex |
| 1 | rs2776819 | 23143623 | T | intron | *LUZP1* | *HTR1D* | cortex |
| 1 | rs61777102 | 23216859 | C | intron | *HTR1D* | *HTR1D* | cortex |
| 1 | rs9787309 | 23225613 | G | regulatory region | - | *HTR1D* | cortex |
| 1 | rs12143690 | 23230924 | A | intergenic | - | *HTR1D* | cortex |
| 1 | rs9662343 | 23247558 | C | downstream gene | - | *HTR1D* | cortex |
| 1 | rs12565271 | 23262721 | G | intergenic | - | *HTR1D* | cortex |
| 1 | rs10799794 | 23374625 | A | upstream gene | *ZNF436* | *HTR1D* | cortex |
| 1 | rs4648892 | 23393692 | T | intron | *TCEA3* | *HTR1D* | frontal cortex |
| 1 | rs6424116 | 23832285 | A | intron | *HMGCL* | *PITHD1* | whole blood |
| 1 | rs12067642 | 45461845 | G | upstream gene | *TESK2* | *FAAH* | whole blood |
| 1 | rs2883968 | 45520188 | G | intron | *PRDX1* | *FAAH* | whole blood |
| 1 | rs11211139 | 45550079 | G | upstream gene | *AKR1A1* | *FAAH* | whole blood |
| 1 | rs2991976 | 45582817 | T | upstream gene | *NASP* | *FAAH* | whole blood |
| 1 | rs11590549 | 45620657 | C | downstream gene | *NASP* | *FAAH* | whole blood |
| 1 | rs6699418 | 45634509 | G | intron | *GPBP1L1* | *FAAH* | whole blood |
| 1 | rs6665808 | 45696611 | C | intron | *IPP* | *FAAH* | whole blood |
| 1 | rs6700974 | 45906063 | A | intron | *MAST2* | *FAAH* | whole blood |
| 1 | rs4420029 | 45959828 | A | intron | *MAST2* | *FAAH* | whole blood |

| | | | | | | | |
|---|---|---|---|---|---|---|---|
| 1 | rs785504 | 46130024 | T | intron | *PIK3R3* | *FAAH* | whole blood |
| 1 | rs3845301 | 46169537 | A | intron | *PIK3R3* | *FAAH* | whole blood |
| 1 | rs12131635 | 46170223 | T | upstream gene | *TSPAN1* | *FAAH* | whole blood |
| 1 | rs12141928 | 46212246 | A | intron | *LURAP1* | *FAAH* | whole blood |
| 1 | rs9793263 | 46256717 | A | intron | *RAD54L* | *FAAH* | whole blood |
| 1 | rs12125573 | 46269120 | T | intron | *RAD54L* | *FAAH* | whole blood |
| 1 | rs12217016 | 46335651 | C | intergenic | - | *FAAH* | caudate |
| 1 | rs12562440 | 46348918 | A | intron | *NSUN4* | *FAAH* | whole blood |
| 1 | rs6687917 | 46432666 | A | upstream gene | *FAAHP1* | *FAAH* | whole blood |
| 1 | rs942257 | 46450847 | G | upstream gene | *LINC01398* | *FAAH* | whole blood |
| 1 | rs12410520 | 202174346 | C | intron | *PTPRVP* | *ADIPOR1* | whole blood |
| 3 | rs35681 | 10287693 | C | intron | *GHRL* | - | - |
| 3 | rs35680 | 10288880 | T | intron | *GHRL* | - | - |
| 3 | rs26802 | 10290681 | T | intron | *GHRL* | - | - |
| 3 | rs26311 | 10291242 | C | 5-prime UTR | *GHRL* | - | - |
| 3 | rs27498 | 10292337 | G | intron | *GHRL* | - | - |
| 3 | rs113910445 | 11147938 | T | intron | *HRH1* | - | - |
| 3 | rs748998 | 11149215 | G | intron | *HRH1* | - | - |
| 3 | rs6799878 | 11159146 | A | intron | *HRH1* | - | - |
| 3 | rs7615837 | 11180344 | A | intron | *HRH1* | - | - |
| 3 | rs12489945 | 11180721 | G | intron | *HRH1* | - | - |
| 3 | rs11128542 | 11183027 | C | intron | *HRH1* | - | - |
| 3 | rs347626 | 11188511 | C | intron | *HRH1* | - | - |
| 3 | rs4684059 | 11191093 | G | intron | *HRH1* | - | - |
| 3 | rs7639145 | 11192499 | G | intron | *HRH1* | - | - |
| 3 | rs35282657 | 11202835 | G | intron | *HRH1* | - | - |
| 3 | rs346067 | 11210409 | C | intron | *HRH1* | - | - |
| 3 | rs6773737 | 11223636 | C | upstream gene | *HRH1* | - | - |
| 3 | rs73812409 | 11247579 | A | intron | *HRH1* | - | - |
| 3 | rs2606731 | 11249929 | C | upstream gene | *HRH1* | - | - |
| 3 | rs1018111 | 11257836 | G | intron | *HRH1* | - | - |
| 3 | rs7632193 | 114129346 | A | intron | *DRD3* | - | - |
| 3 | rs324035 | 114150007 | C | intron | *DRD3* | - | - |
| 3 | rs1800828 | 114172702 | C | intron | *DRD3* | - | - |
| 3 | rs7638876 | 114175453 | C | intron | *DRD3* | - | - |
| 3 | rs461602 | 10212139 | A | intron | *IRAK2* | *GHRL* | cortex |
| 3 | rs35684 | 10285002 | A | downstream gene | *TATDN2* | *GHRL* | cortex |
| 3 | rs41147 | 10326418 | A | 3-prime UTR | *ATP2B2* | *GHRL* | whole blood |

http://doi.org/10.47626/1516-4446-2024-3608


| | | | | | | | |
|---|---|---|---|---|---|---|---|
| 3 | rs1719571 | 10327496 | A | 3-prime UTR | *ATP2B2* | *GHRL* | whole blood |
| 4 | rs356203 | 89744890 | T | intron | *SNCA* | - | - |
| 4 | rs3910105 | 89761420 | A | intron | *SNCA* | - | - |
| 4 | rs3910104 | 89769597 | C | intron | *SNCA* | - | - |
| 4 | rs2583963 | 89805937 | T | intron | *SNCA* | - | - |
| 4 | rs7356297 | 89825178 | T | intron | *SNCA* | - | - |
| 4 | rs2245804 | 89836797 | T | intron | *SNCA* | - | - |
| 4 | rs2583960 | 89803718 | A | intron | *SNCA* | *SNCA* | whole blood |
| 4 | rs10005233 | 89822180 | T | intron | *SNCA* | *SNCA* | whole blood |
| 4 | rs3113355 | 89824194 | T | intron | *SNCA* | *SNCA* | whole blood |
| 4 | rs920624 | 89827044 | A | intron | *SNCA* | *SNCA* | whole blood |
| 4 | rs2583990 | 89850348 | G | intergenic | - | *SNCA* | whole blood |
| 4 | rs1442153 | 89854568 | T | intergenic | - | *SNCA* | whole blood |
| 4 | rs11725564 | 89856889 | G | intergenic | - | *SNCA* | whole blood |
| 4 | rs6841352 | 89860504 | G | intergenic | - | *SNCA* | whole blood |
| 5 | rs6884105 | 159921436 | G | intron | *ADRA1B* | - | - |
| 5 | rs62377666 | 159924942 | A | intron | *ADRA1B* | - | - |
| 5 | rs11952941 | 159927768 | T | intron | *ADRA1B* | - | - |
| 5 | rs34235993 | 159934421 | T | intron | *ADRA1B* | - | - |
| 5 | rs686 | 175441697 | G | 3-prime UTR | *DRD1* | - | - |
| 5 | rs10080093 | 63887486 | A | intergenic | - | *HTR1A* | cortex |
| 6 | rs4716053 | 16249603 | G | intron | *GMPR* | - | - |
| 6 | rs6926552 | 16290631 | T | intron | *GMPR* | - | - |
| 6 | rs6723 | 16295358 | C | downstream gene | *ATXN1* | - | - |
| 6 | rs9465733 | 20409544 | C | intron | *E2F3* | - | - |
| 6 | rs17569598 | 20414567 | C | intron | *E2F3* | - | - |
| 6 | rs942043 | 20427953 | T | intron | *E2F3* | - | - |
| 6 | rs9465741 | 20428201 | C | intron | *E2F3* | - | - |
| 6 | rs16883843 | 20461201 | A | intron | *E2F3* | - | - |
| 6 | rs7747931 | 20482180 | G | intron | *E2F3* | - | - |
| 6 | rs4134958 | 20486275 | G | intron | *E2F3* | - | - |
| 6 | rs16878041 | 86956775 | C | intron | *HTR1E* | - | - |
| 6 | rs4707336 | 86982199 | C | intron | *HTR1E* | - | - |
| 6 | rs3734198 | 89254573 | A | 3-prime UTR | *GABRR2* | - | - |
| 6 | rs282131 | 89256091 | C | 3-prime UTR | *GABRR2* | - | - |
| 6 | rs4706338 | 89263333 | A | intron | *GABRR2* | - | - |
| 6 | rs9294426 | 89264859 | G | intron | *GABRR2* | - | - |
| 6 | rs9451192 | 89266653 | G | intron | *GABRR2* | - | - |

| | | | | | | | |
|---|---|---|---|---|---|---|---|
| 6 | rs4707535 | 89268187 | C | intron | *GABRR2* | - | - |
| 6 | rs9444682 | 89275624 | G | intron | *GABRR2* | - | - |
| 6 | rs12207013 | 89280420 | C | intron | *GABRR2* | - | - |
| 6 | rs3777525 | 89282019 | A | intron | *GABRR2* | - | - |
| 6 | rs4707536 | 89295842 | C | intron | *GABRR2* | - | - |
| 6 | rs2016843 | 89297772 | G | intron | *GABRR2* | - | - |
| 6 | rs3777519 | 89298306 | C | intron | *GABRR2* | - | - |
| 6 | rs3777518 | 89298641 | G | intron | *GABRR2* | - | - |
| 6 | rs3777516 | 89302744 | C | intron | *GABRR2* | - | - |
| 6 | rs9294431 | 89309777 | A | intron | *GABRR2* | - | - |
| 6 | rs9464964 | 16932552 | C | intron | - | *GMPR* | frontal cortex |
| 6 | rs4713409 | 31013422 | T | intron | *MUC22* | *TNF* | hippocampus |
| 6 | rs6910636 | 31018016 | C | intron | *MUC22* | *TNF* | hippocampus |
| 6 | rs1570028 | 89302716 | A | intron | *GABRR2* | *GABRR2* | whole blood |
| 6 | rs3822872 | 89310729 | T | intron | *GABRR2* | *GABRR2* | whole blood |
| 6 | rs10944441 | 89311822 | G | intron | *GABRR2* | *GABRR2* | whole blood |
| 6 | rs2236204 | 89315393 | G | upstream gene | *GABRR2* | *GABRR2* | whole blood |
| 6 | rs7757650 | 89320926 | T | intergenic | - | *GABRR2* | whole blood |
| 7 | rs274615 | 86649621 | A | intron | *GRM3* | - | - |
| 7 | rs2282960 | 86655951 | C | intron | *GRM3* | - | - |
| 7 | rs12669901 | 86678472 | G | intron | *GRM3* | - | - |
| 7 | rs724224 | 86696304 | G | intron | *GRM3* | - | - |
| 7 | rs802478 | 86697364 | C | intron | *GRM3* | - | - |
| 7 | rs1012973 | 86704812 | G | intron | *GRM3* | - | - |
| 7 | rs2189812 | 86719683 | G | intron | *GRM3* | - | - |
| 7 | rs6943659 | 86766610 | T | intron | *GRM3* | - | - |
| 7 | rs720025 | 86781542 | T | intron | *GRM3* | - | - |
| 7 | rs2237562 | 86792916 | T | intron | *GRM3* | - | - |
| 7 | rs2237564 | 86816627 | T | intron | *GRM3* | - | - |
| 7 | rs2282966 | 86846351 | G | intron | *GRM3* | - | - |
| 7 | rs7789655 | 86851697 | T | intron | *GRM3* | - | - |
| 7 | rs4148737 | 87541836 | T | intron | *ABCB1* | - | - |
| 7 | rs13237132 | 87562353 | C | intron | *ABCB1* | - | - |
| 7 | rs10264990 | 87573299 | T | intron | *ABCB1* | - | - |
| 7 | rs1202179 | 87574963 | T | intron | *ABCB1* | - | - |
| 7 | rs11763872 | 87587899 | T | intron | *ABCB1* | - | - |
| 7 | rs11761556 | 128257016 | C | 3-prime UTR | *LEP* | - | - |
| 7 | rs1805123 | 150948446 | T | missense | *KCNH2* | - | - |

http://doi.org/10.47626/1516-4446-2024-3608


| | | | | | | | |
|---|---|---|---|---|---|---|---|
| 7 | rs882156 | 150956248 | C | intron | *KCNH2* | - | - |
| 7 | rs4725386 | 150963151 | C | intron | *KCNH2* | - | - |
| 7 | rs3800779 | 150974126 | C | intron | *KCNH2* | - | - |
| 7 | rs3807370 | 150976226 | G | intron | *KCNH2* | - | - |
| 8 | rs10088810 | 22064239 | G | intron | *DMTN* | - | - |
| 8 | rs4436136 | 26783888 | T | intron | *ADRA1A* | - | - |
| 8 | rs12680333 | 26801849 | C | intron | *ADRA1A* | - | - |
| 8 | rs13261054 | 26809528 | C | intron | *ADRA1A* | - | - |
| 8 | rs4732661 | 26812758 | G | intron | *ADRA1A* | - | - |
| 8 | rs4732900 | 26820138 | C | intron | *ADRA1A* | - | - |
| 8 | rs498989 | 26833832 | C | intron | *ADRA1A* | - | - |
| 8 | rs6986495 | 26833848 | T | intron | *ADRA1A* | - | - |
| 8 | rs2322333 | 26837738 | G | intron | *ADRA1A* | - | - |
| 8 | rs10503800 | 26838100 | C | intron | *ADRA1A* | - | - |
| 8 | rs571742 | 26839196 | G | intron | *ADRA1A* | - | - |
| 8 | rs558455 | 26848064 | G | intron | *ADRA1A* | - | - |
| 8 | rs476631 | 26848942 | T | intron | *ADRA1A* | - | - |
| 8 | rs544215 | 26854511 | A | intron | *ADRA1A* | - | - |
| 8 | rs11782159 | 26855464 | A | intron | *ADRA1A* | - | - |
| 8 | rs13278849 | 26857357 | A | intron | *ADRA1A* | - | - |
| 8 | rs573514 | 26863764 | A | intron | *ADRA1A* | - | - |
| 8 | rs7386762 | 22069949 | G | intron | *DMTN* | *DMTN* | liver |
| 8 | rs2086836 | 22087451 | G | downstream gene | *DMTN* | *DMTN* | liver |
| 8 | rs2309233 | 22088007 | C | upstream gene | *FAM160B2* | *DMTN* | liver |
| 8 | rs10088604 | 22156798 | C | 5-prime UTR | *LGI3* | *DMTN* | liver |
| 9 | rs913991 | 4493141 | A | intron | *SLC1A1* | - | - |
| 9 | rs10814991 | 4495254 | C | intron | *SLC1A1* | - | - |
| 9 | rs10814992 | 4497069 | A | intron | *SLC1A1* | - | - |
| 9 | rs10814993 | 4497428 | A | intron | *SLC1A1* | - | - |
| 9 | rs10739062 | 4502848 | C | intron | *SLC1A1* | - | - |
| 9 | rs2039291 | 4507705 | A | intron | *SLC1A1* | - | - |
| 9 | rs10117861 | 4518484 | A | intron | *SLC1A1* | - | - |
| 9 | rs10118904 | 4519911 | A | intron | *SLC1A1* | - | - |
| 9 | rs10118909 | 4519960 | T | intron | *SLC1A1* | - | - |
| 9 | rs7024664 | 4524371 | T | intron | *SLC1A1* | - | - |
| 9 | rs10758629 | 4528816 | T | intron | *SLC1A1* | - | - |
| 9 | rs7468917 | 4541753 | C | intron | *SLC1A1* | - | - |
| 9 | rs6476876 | 4548122 | C | intron | *SLC1A1* | - | - |

| | | | | | | | |
|---|---|---|---|---|---|---|---|
| 9 | rs28420685 | 4555209 | C | intron | *SLC1A1* | - | - |
| 9 | rs7861103 | 4565399 | T | intron | *SLC1A1* | - | - |
| 9 | rs3780413 | 4567353 | G | intron | *SLC1A1* | - | - |
| 9 | rs301979 | 4576851 | C | intron | *SLC1A1* | - | - |
| 9 | rs301435 | 4582843 | T | intron | *SLC1A1* | - | - |
| 9 | rs12551373 | 34092432 | A | intron | *DCAF12* | - | - |
| 9 | rs10971908 | 34092974 | T | intron | *DCAF12* | - | - |
| 9 | rs10971920 | 34110848 | G | intron | *DCAF12* | - | - |
| 9 | rs56157435 | 34111105 | C | intron | *DCAF12* | - | - |
| 9 | rs4879747 | 34118901 | A | intron | *DCAF12* | - | - |
| 9 | rs9792458 | 128000399 | C | intergenic | - | *ST6GALNAC4* | whole blood |
| 10 | rs11498504 | 90765079 | T | intron | *HTR7* | - | - |
| 10 | rs4471341 | 90773525 | G | intron | *HTR7* | - | - |
| 10 | rs12783703 | 90800656 | A | intron | *HTR7* | - | - |
| 10 | rs9420571 | 90829042 | A | intron | *HTR7* | - | - |
| 10 | rs12259062 | 90850288 | G | intron | *HTR7* | - | - |
| 10 | rs1891233 | 90399099 | T | downstream gene | *LINC02653* | *HTR7* | putamen |
| 10 | rs10785957 | 90407355 | T | intron | *LINC02653* | *HTR7* | putamen |
| 10 | rs7079277 | 111122984 | T | intergenic | - | *ADRA2A* | nucleus accumbens |
| 10 | rs10885090 | 111131159 | C | regulatory region | - | *ADRA2A* | nucleus accumbens |
| 10 | rs1335706 | 111155014 | T | intergenic | - | *ADRA2A* | nucleus accumbens |
| 10 | rs7908674 | 111163613 | G | intergenic | - | *ADRA2A* | nucleus accumbens |
| 10 | rs61863016 | 111184995 | C | intergenic | - | *ADRA2A* | caudate |
| 10 | rs1832112 | 111226281 | A | intergenic | - | *ADRA2A* | nucleus accumbens |
| 10 | rs7476362 | 111240559 | A | intergenic | - | *ADRA2A* | cortex |
| 10 | rs12241611 | 111251893 | T | intergenic | - | *ADRA2A* | nucleus accumbens |
| 10 | rs7084296 | 111288465 | A | intergenic | - | *ADRA2A* | nucleus accumbens |
| 11 | rs7124601 | 639273 | C | intron | *DRD4* | - | - |
| 11 | rs28540443 | 639374 | G | intron | *DRD4* | - | - |
| 11 | rs55697087 | 113450489 | C | intron | *DRD2* | - | - |
| 11 | rs10891553 | 113466545 | A | intron | *DRD2* | - | - |
| 11 | rs8176333 | 535649 | C | upstream gene | *LRRC56* | *DRD4* | whole blood |
| 11 | rs12793222 | 554047 | G | missense | *LRRC56* | *DRD4* | cortex |
| 11 | rs736247 | 571316 | T | upstream gene | *MIR210* | *DRD4* | cortex |
| 11 | rs35865896 | 584591 | A | intron | *PHRF1* | *DRD4* | cortex |
| 11 | rs4963128 | 589564 | C | intron | *PHRF1* | *DRD4* | cortex |
| 11 | rs34610235 | 589581 | A | intron | *PHRF1* | *DRD4* | cortex |
| 11 | rs12272314 | 590648 | A | intron | *PHRF1* | *DRD4* | cortex |

http://doi.org/10.47626/1516-4446-2024-3608


| | | | | | | | |
|---|---|---|---|---|---|---|---|
| 11 | rs12421158 | 609888 | T | intron | *PHRF1* | *DRD4* | cortex |
| 11 | rs719802 | 113363957 | C | intron | *TTC12* | *ANKK1* | whole blood |
| 11 | rs10891540 | 113368360 | G | 3-prime UTR | *TTC12* | *ANKK1* | whole blood |
| 11 | rs10891549 | 113407725 | T | downstream gene | *DRD2* | *ANKK1* | whole blood |
| 11 | rs11608185 | 113424254 | T | intron | *DRD2* | *ANKK1* | whole blood |
| 11 | rs4648318 | 113442667 | T | intron | *DRD2* | *ANKK1* | whole blood |
| 12 | rs10863088 | 85881965 | A | intron | *NTS* | - | - |
| 12 | rs10744888 | 117069204 | G | intron | *TESC* | - | - |
| 12 | rs7954608 | 117070532 | C | intron | *TESC* | - | - |
| 12 | rs4767469 | 116889049 | T | regulatory region | - | *TESC* | frontal cortex |
| 12 | rs12320925 | 116899296 | T | intergenic | - | *TESC* | frontal cortex |
| 12 | rs7955009 | 117040777 | C | intron | *TESC* | *TESC* | whole blood |
| 12 | rs3782191 | 117042896 | G | intron | *TESC* | *TESC* | whole blood |
| 12 | rs3782192 | 117058929 | C | intron | *TESC* | *TESC* | whole blood |
| 13 | rs7997012 | 46837850 | G | intron | *HTR2A* | - | - |
| 13 | rs9567737 | 46847131 | T | intron | *HTR2A* | - | - |
| 13 | rs1923885 | 46848951 | T | upstream gene | *HTR2A-AS1* | - | - |
| 13 | rs7330461 | 46849430 | A | upstream gene | *HTR2A-AS1* | - | - |
| 13 | rs1745837 | 46850677 | T | upstream gene | *HTR2A-AS1* | - | - |
| 13 | rs9567739 | 46850809 | G | upstream gene | *HTR2A-AS1* | - | - |
| 13 | rs2760351 | 46856180 | G | exon | *HTR2A-AS1* | - | - |
| 13 | rs2246127 | 46856344 | A | downstream gene | *HTR2A-AS1* | - | - |
| 13 | rs4942578 | 46858475 | G | downstream gene | *HTR2A-AS1* | - | - |
| 13 | rs4942579 | 46859709 | C | downstream gene | *HTR2A-AS1* | - | - |
| 13 | rs4941574 | 46890939 | G | intron | *HTR2A* | - | - |
| 13 | rs2070040 | 46893491 | G | intron | *HTR2A* | - | - |
| 13 | rs2025736 | 46120765 | A | downstream gene | *CPB2-AS1* | *HTR2A* | liver |
| 13 | rs6561332 | 46845685 | G | intron | *HTR2A* | *HTR2A* | caudate |
| 14 | rs1952437 | 54856732 | C | intron | *GCH1* | - | - |
| 14 | rs8018688 | 54898663 | A | intron | *GCH1* | - | - |
| 14 | rs2896288 | 94615407 | G | intron | *SERPINA3* | - | - |
| 14 | rs2268336 | 94616779 | T | intron | *SERPINA3* | - | - |
| 14 | rs910350 | 94621578 | A | intron | *SERPINA3* | - | - |
| 14 | rs2498794 | 104778914 | A | intron | *AKT1* | - | - |
| 14 | rs2494746 | 104791382 | G | intron | *AKT1* | - | - |
| 14 | rs2145945 | 54837362 | G | downstream gene | *GCH1* | *GCH1* | whole blood |
| 14 | rs28417208 | 54887070 | T | intron | *GCH1* | *GCH1* | whole blood |
| 14 | rs8010570 | 54919944 | G | intergenic | - | *GCH1* | whole blood |

http://doi.org/10.47626/1516-4446-2024-3608


| | | | | | | | |
|---|---|---|---|---|---|---|---|
| 15 | rs28564957 | 78613765 | A | intron | *CHRNA3* | - | - |
| 15 | rs8041121 | 74437951 | G | upstream gene | *SEMA7A* | *CYP1A1* | nucleus accumbens |
| 15 | rs8037641 | 74541182 | T | upstream gene | *ARID3B* | *CYP1A1* | nucleus accumbens |
| 15 | rs11639224 | 78461029 | A | intron | *IREB2* | *CHRNA3* | caudate |
| 15 | rs11636131 | 78529264 | C | intron | *HYKK* | *CHRNA3* | caudate |
| 15 | rs1847530 | 78531689 | G | intron | *HYKK* | *CHRNA3* | caudate |
| 15 | rs55690619 | 78541270 | G | intron | *PSMA4* | *CHRNA3* | caudate |
| 15 | rs77434238 | 78544384 | C | intron | *PSMA4* | *CHRNA3* | caudate |
| 15 | rs12907966 | 78550709 | C | 3-prime UTR | *PSMA4* | *CHRNA3* | caudate |
| 15 | rs28395178 | 78558216 | G | intergenic | - | *CHRNA3* | nucleus accumbens |
| 15 | rs601079 | 78577237 | A | intron | *CHRNA5* | *CHRNA3* | caudate |
| 15 | rs8192478 | 78618120 | A | intron | *CHRNA3* | *CHRNA3* | caudate |
| 15 | rs8040868 | 78618839 | T | synonymous | *CHRNA3* | *CHRNA3* | putamen |
| 15 | rs1878399 | 78619661 | C | downstream gene | *CHRNB4* | *CHRNA3* | caudate |
| 15 | rs13329271 | 78621888 | A | downstream gene | *CHRNB4* | *CHRNA3* | caudate |
| 15 | rs1316971 | 78638168 | G | intron | *CHRNB4* | *CHRNA3* | caudate |
| 15 | rs11857532 | 78675926 | T | intron | *CHRNB4* | *CHRNA3* | nucleus accumbens |
| 16 | rs2076452 | 1819325 | T | intron | *HAGH* | - | - |
| 16 | rs11248882 | 1507089 | A | intron | *TELO2* | *HAGH* | nucleus accumbens |
| 16 | rs2667684 | 1600945 | G | intron | *IFT140* | *HAGH* | substantia nigra |
| 16 | rs7204263 | 1628334 | G | intron | *CRAMP1* | *HAGH* | substantia nigra |
| 16 | rs9927962 | 1753533 | C | intron | *MAPK8IP3* | *HAGH* | substantia nigra |
| 16 | rs1178437 | 1781911 | C | upstream gene | *NUBP2* | *HAGH* | putamen |
| 16 | rs2745170 | 1785230 | T | intron | *NUBP2* | *HAGH* | substantia nigra |
| 16 | rs182939 | 1789376 | C | downstream gene | *IGFALS* | *HAGH* | substantia nigra |
| 16 | rs4786635 | 1812723 | G | intron | *HAGH* | *HAGH* | substantia nigra |
| 16 | rs238682 | 1829096 | A | downstream gene | *MEIOB* | *HAGH* | nucleus accumbens |
| 16 | rs5006376 | 1855200 | T | intron | *MEIOB* | *HAGH* | caudate |
| 16 | rs11862015 | 1881652 | T | intron | *MEIOB* | *HAGH* | nucleus accumbens |
| 16 | rs337285 | 1911673 | G | missense | *HS3ST6* | *HAGH* | caudate |
| 16 | rs344366 | 1926151 | A | intergenic | - | *HAGH* | substantia nigra |
| 16 | rs6600183 | 1934934 | C | downstream gene | *MSRB1* | *HAGH* | caudate |
| 17 | rs8066602 | 30196067 | C | 3-prime UTR | *SLC6A4* | - | - |
| 17 | rs34388196 | 30207451 | A | intron | *SLC6A4* | - | - |
| 17 | rs140701 | 30211514 | C | intron | *SLC6A4* | - | - |
| 17 | rs4583306 | 30211697 | A | intron | *SLC6A4* | - | - |
| 17 | rs4404139 | 73218448 | C | intron | *FAM104A* | - | - |
| 17 | rs11550611 | 82079001 | G | 3-prime UTR | *FASN* | - | - |

http://doi.org/10.47626/1516-4446-2024-3608

http://doi.org/10.47626/1516-4446-2024-3608


| | | | | | | | |
|---|---|---|---|---|---|---|---|
| 17 | rs8072894 | 8409351 | T | upstream gene | *NDEL1* | *NDEL1* | whole blood |
| 17 | rs8081022 | 8414434 | G | intron | *NDEL1* | *NDEL1* | whole blood |
| 17 | rs4791314 | 8424562 | C | intron | *NDEL1* | *NDEL1* | whole blood |
| 17 | rs1972989 | 73191134 | A | upstream gene | *COG1* | *FAM104A* | whole blood |
| 17 | rs1052706 | 73196524 | G | synonymous | *COG1* | *FAM104A* | whole blood |
| 17 | rs9901287 | 73218611 | G | intron | *FAM104A* | *FAM104A* | whole blood |
| 17 | rs35855827 | 73234793 | A | intron | *C17orf80* | *FAM104A* | whole blood |
| 17 | rs745142 | 73236742 | G | missense | *C17orf80* | *FAM104A* | whole blood |
| 17 | rs9900555 | 73256884 | G | intron | *CPSF4L* | *FAM104A* | whole blood |
| 17 | rs72861776 | 82041417 | T | upstream gene | *DCXR* | *FASN* | whole blood |
| 17 | rs7215515 | 82077662 | C | downstream gene | *FASN* | *FASN* | whole blood |
| 17 | rs17848939 | 82085473 | C | intron | *FASN* | *FASN* | whole blood |
| 17 | rs2229422 | 82085863 | A | synonymous | *FASN* | *FASN* | whole blood |
| 17 | rs17848934 | 82096499 | C | intron | *FASN* | *FASN* | whole blood |
| 17 | rs35158616 | 82136604 | G | intron | *CCDC57* | *FASN* | whole blood |
| 17 | rs10163482 | 82152518 | A | downstream gene | *CCDC57* | *FASN* | whole blood |
| 17 | rs9303024 | 82226192 | G | upstream gene | *SLC16A3* | *FASN* | whole blood |
| 17 | rs3176828 | 82232138 | C | upstream gene | *SLC16A3* | *FASN* | whole blood |
| 17 | rs3176827 | 82232300 | G | 5-prime UTR | *SLC16A3* | *FASN* | whole blood |
| 17 | rs35121878 | 82232895 | T | intron | *SLC16A3* | *FASN* | whole blood |
| 17 | rs7502358 | 82233451 | C | intron | *SLC16A3* | *FASN* | whole blood |
| 17 | rs2256833 | 82731791 | C | downstream gene | *FN3KRP* | *FASN* | hypothalamus |
| 18 | rs4890876 | 77020131 | T | upstream gene | *MBP* | - | - |
| 18 | rs8096433 | 77021017 | T | upstream gene | *MBP* | - | - |
| 18 | rs7407423 | 77029498 | C | intron | *MBP* | - | - |
| 18 | rs470276 | 77031244 | C | intron | *MBP* | - | - |
| 18 | rs4890879 | 77032984 | T | intron | *MBP* | - | - |
| 18 | rs11661965 | 77036046 | T | intron | *MBP* | - | - |
| 18 | rs470190 | 77043280 | G | intron | *MBP* | - | - |
| 18 | rs470212 | 77047423 | G | intron | *MBP* | - | - |
| 18 | rs56060144 | 77057043 | C | intron | *MBP* | - | - |
| 18 | rs9676181 | 77057219 | A | intron | *MBP* | - | - |
| 18 | rs56014377 | 77059083 | C | intron | *MBP* | - | - |
| 18 | rs8095585 | 77061311 | G | intron | *MBP* | - | - |
| 18 | rs4890888 | 77067910 | G | intron | *MBP* | - | - |
| 18 | rs557659 | 77074267 | A | intron | *MBP* | - | - |
| 18 | rs4890891 | 77099973 | C | intron | *MBP* | - | - |
| 18 | rs3813079 | 77101215 | G | intron | *MBP* | - | - |

| | | | | | | | |
|---|---|---|---|---|---|---|---|
| 18 | rs1629089 | 77113812 | C | intron | *MBP* | - | - |
| 18 | rs1789092 | 77115050 | C | intron | *MBP* | - | - |
| 18 | rs1789086 | 77117427 | C | intron | *MBP* | - | - |
| 18 | rs34783785 | 77117760 | G | intron | *MBP* | - | - |
| 18 | rs1450190 | 77125700 | G | intron | *MBP* | - | - |
| 18 | rs1375967 | 77131414 | C | intron | *MBP* | - | - |
| 20 | rs7272062 | 31671342 | A | intron | *BCL2L1* | - | - |
| 20 | rs6058421 | 31707210 | G | intron | *BCL2L1* | - | - |
| 20 | rs6120835 | 31602250 | T | upstream gene | *ID1* | *BCL2L1* | frontal cortex |
| 22 | rs1544325 | 19944145 | G | upstream gene | *TXNRD2* | - | - |
| 22 | rs174682 | 19948084 | G | upstream gene | *COMT* | - | - |
| 22 | rs5993883 | 19950115 | G | upstream gene | *COMT* | - | - |
| 22 | rs165767 | 19957037 | G | intron | *COMT* | - | - |
| 22 | rs174695 | 19959946 | C | intron | *COMT* | - | - |
| 22 | rs2239393 | 19962905 | A | intron | *COMT* | - | - |
| 22 | rs9306234 | 19965665 | A | intron | *COMT* | - | - |
| 22 | rs12628032 | 19980457 | C | intron | *ARVCF* | - | - |
| 22 | rs756653 | 19989322 | G | intron | *ARVCF* | - | - |
| 22 | rs2079701 | 20004492 | A | intron | *ARVCF* | - | - |
| 22 | rs5998516 | 32496320 | A | intron | *FBXO7* | - | - |
| 22 | rs4485648 | 19931882 | T | intron | *TXNRD2* | *COMT* | whole blood |
| 22 | rs4680 | 19963748 | G | missense | *COMT* | *ARVCF* | whole blood |
| 22 | rs165849 | 19971146 | A | downstream gene | *COMT* | *ARVCF* | whole blood |
| 22 | rs9606210 | 20017735 | T | upstream gene | *ARVCF* | *ARVCF* | whole blood |
| 22 | rs7288316 | 20039002 | A | intron | *TANGO2* | *ARVCF* | whole blood |
| 22 | rs419135 | 20062878 | T | intron | *TANGO2* | *ARVCF* | whole blood |
| 22 | rs391310 | 20067559 | G | downstream gene | *TANGO2* | *ARVCF* | whole blood |
| 22 | rs595224 | 20154469 | G | upstream gene | *CCDC188* | *ARVCF* | whole blood |
| 22 | rs5994578 | 32500603 | T | downstream gene | *FBXO7* | *FBXO7* | hippocampus |
| 22 | rs4820431 | 41153917 | C | intron | *EP300* | *CYP2D6* | cortex |
| 22 | rs9607779 | 41170819 | G | intron | *EP300* | *CYP2D6* | cortex |
| 22 | rs139492 | 41232898 | G | downstream gene | *L3MBTL2* | *CYP2D6* | cortex |
| 22 | rs139493 | 41236075 | C | downstream gene | *L3MBTL2* | *CYP2D6* | whole blood |
| 22 | rs7289932 | 41307800 | A | intron | *ZC3H7B* | *CYP2D6* | cortex |
| 22 | rs2273071 | 41394496 | C | intron | *TEF* | *CYP2D6* | whole blood |
| 22 | rs2228314 | 41880738 | G | missense | *SREBF2* | *CYP2D6* | whole blood |
| 22 | rs2267442 | 41884357 | A | intron | *SREBF2* | *CYP2D6* | whole blood |
| 22 | rs6002548 | 41941036 | T | intron | *CENPM* | *CYP2D6* | whole blood |

| | | | | | | | |
|---|---|---|---|---|---|---|---|
| 22 | rs5751182 | 41949177 | A | upstream gene | *CENPM* | *CYP2D6* | whole blood |
| 22 | rs5996096 | 41965332 | G | upstream gene | *SEPTIN3* | *CYP2D6* | whole blood |
| 22 | rs2899350 | 41967404 | A | upstream gene | *SEPTIN3* | *CYP2D6* | whole blood |
| 22 | rs4822076 | 41968053 | T | upstream gene | *SEPTIN3* | *CYP2D6* | whole blood |
| 22 | rs5758536 | 41992071 | C | intron | *SEPTIN3* | *CYP2D6* | whole blood |
| 22 | rs133317 | 42006382 | G | intron | *WBP2NL* | *CYP2D6* | whole blood |
| 22 | rs133332 | 42015660 | C | intron | *WBP2NL* | *CYP2D6* | whole blood |
| 22 | rs133344 | 42023429 | A | intron | *WBP2NL* | *CYP2D6* | whole blood |
| 22 | rs133346 | 42024851 | G | intron | *WBP2NL* | *CYP2D6* | whole blood |
| 22 | rs5758573 | 42072246 | T | upstream gene | *PHETA2* | *CYP2D6* | whole blood |
| 22 | rs34385013 | 42125924 | G | downstream gene | *CYP2D6* | *CYP2D6* | whole blood |
| 22 | rs4993393 | 42138066 | A | downstream gene | *CYP2D7* | *CYP2D6* | whole blood |
| 22 | rs2743451 | 42138673 | T | downstream gene | *CYP2D7* | *CYP2D6* | whole blood |
| 22 | rs2070904 | 42150456 | T | intron | *CYP2D8P* | *CYP2D6* | whole blood |
| 22 | rs2743465 | 42151748 | G | intron | *CYP2D8P* | *CYP2D6* | whole blood |
| 22 | rs760648 | 42175022 | G | intron | *TCF20* | *CYP2D6* | whole blood |
| 22 | rs2070116 | 42210251 | C | synonymous | *TCF20* | *CYP2D6* | whole blood |
| 22 | rs5758660 | 42227712 | C | intron | *TCF20* | *CYP2D6* | whole blood |
| 22 | rs134886 | 42277850 | G | exon | *OGFRP1* | *CYP2D6* | whole blood |
| 22 | rs134893 | 42281309 | C | downstream gene | *OGFRP1* | *CYP2D6* | whole blood |
| 22 | rs86669 | 42284794 | C | upstream gene | *TCF20* | *CYP2D6* | whole blood |
| 22 | rs134900 | 42287337 | G | upstream gene | *TCF20* | *CYP2D6* | whole blood |
| 22 | rs9611766 | 42292633 | G | intron | *TCF20* | *CYP2D6* | whole blood |
| 22 | rs6002673 | 42295290 | A | intron | *TCF20* | *CYP2D6* | whole blood |
| 22 | rs4820459 | 42302956 | A | intron | *TCF20* | *CYP2D6* | whole blood |
| 22 | rs4822105 | 42302960 | T | intron | *TCF20* | *CYP2D6* | whole blood |
| 22 | rs4822108 | 42309864 | C | intron | *TCF20* | *CYP2D6* | whole blood |
| 22 | rs2413685 | 42321584 | G | intron | *TCF20* | *CYP2D6* | whole blood |
| 22 | rs2092175 | 42383746 | A | 3-prime UTR | *NFAM1* | *CYP2D6* | whole blood |

AGE = altered gene expression (eQTL); n. accumbens: nucleus accumbens; SNV ID = identification of single nucleotide variation.
Brain regions: cortex, nucleus accumbens, substantia nigra, caudate, frontal cortex, hippocampus, putamen, and hypothalamus.
Annotation of 406 SNVs selected by importance in the hybrid prediction model with the highest balanced accuracy.
Position: GRCh38/hg38 was used as the reference.

**Supplementary Table S6** Clinical and genetic predictors selected

| Algorithm | n | Number of SNVs | Clinical predictors |
|---|---|---|---|
| RF | 101 | 406 | CGI, DUP, age, and cannabis use |
| SVM RBF | 100 | 428 | DUP, migration, cannabis, and smoking |
| kNN | 109 | 399 | CGI, age, and cannabis use |

CGI = Clinical Global Impression-Severity; DUP = duration of untreated psychosis; RF = random forests; SNV = single-nucleotide variants; SVM RBF = support vector machine radial basis function; kNN: k-nearest neighbors.

**Supplementary Figure S2** Genomic regions of selected genetic variants

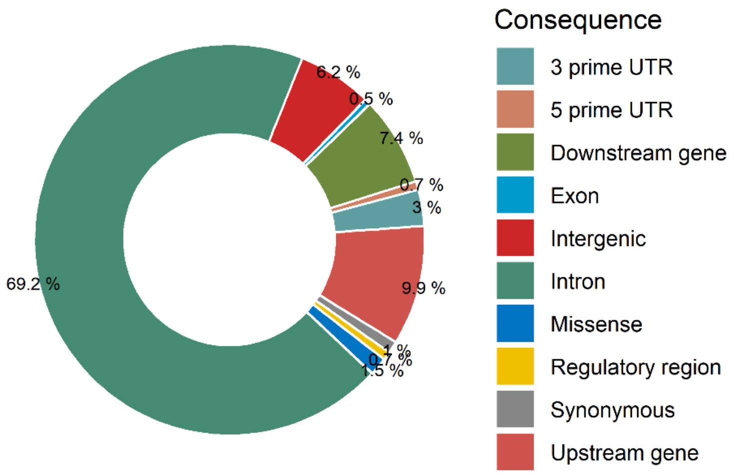


UTR = untranslated region.
Information on the genomic regions and consequences of the variants was obtained using variant effect predictor, an ensemble tool.

**Supplementary Figure S3** Number of eQTLs by gene and tissue

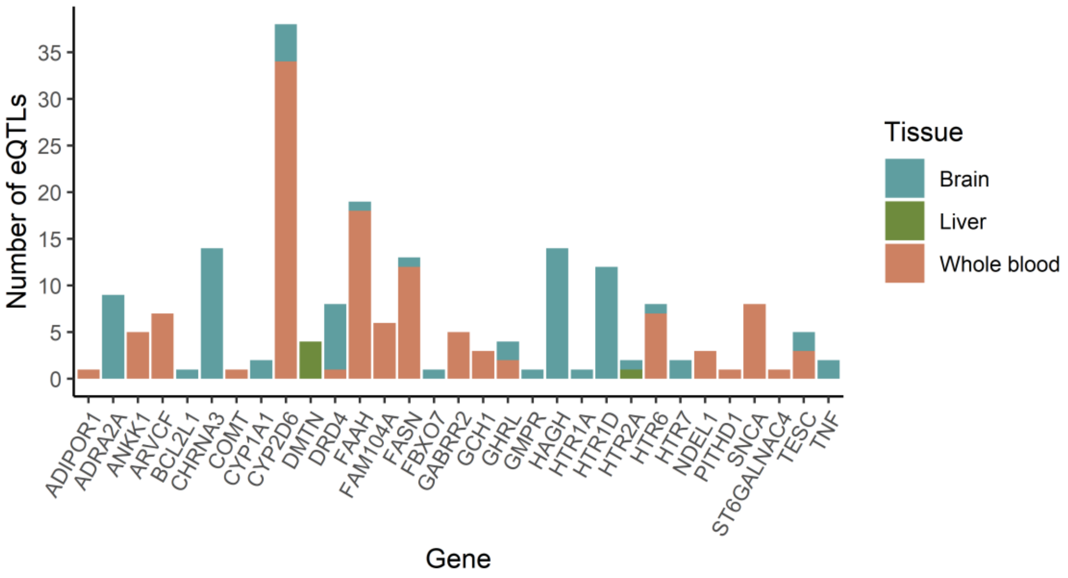


Tissue: tissues in which the single-nucleotide variant regulates the expression of a specific gene, i.e., whole blood, caudate nucleus, brain (cortex), anterior cingulate cortex, brain (frontal cortex), nucleus accumbens, putamen, liver, hippocampus, substantia nigra, amygdala, and hypothalamus. Gene expression altered by eQTLs in brain tissue, whole blood, and liver.
